# SOSTDC1 is down-regulated in non-small cell lung cancer and contributes to cancer cell proliferation

**DOI:** 10.1186/s13578-016-0091-9

**Published:** 2016-04-14

**Authors:** Lei Liu, Shanshan Wu, Yi Yang, Junchao Cai, Xun Zhu, Jueheng Wu, Mengfeng Li, Hongyu Guan

**Affiliations:** Key Laboratory of Tropical Disease Control, Ministry of Education, Sun Yat-sen University, Guangzhou, 510080 Guangdong China; Department of Microbiology, Zhongshan School of Medicine, Sun Yat-sen University, Guangzhou, 510080 Guangdong China; Department of Pharmacology, Zhongshan School of Medicine, Sun Yat-sen University, Guangzhou, 510080 Guangdong China; Department of Endocrinology and Diabetes Center, The First Affiliated Hospital of Sun Yat-sen University, 58 Zhongshan Road II, Guangzhou, 510080 Guangdong China

**Keywords:** SOSTDC1, Non-small cell lung cancer, Proliferation, p21Cip, p27Kip

## Abstract

**Background:**

Non-small cell lung cancer (NSCLC) is the most commonly diagnosed and fatal cancer worldwide. Sclerostin domain containing protein 1 (SOSTDC1) has been found to be tumor-suppressive in several types of cancers. However, the expression level and biological functions of SOSTDC1 in NSCLC remain unknown. Our current study aimed to identify the biological significance of SOSTDC1 in NSCLC.

**Results:**

We found that SOSTDC1 was significantly down-regulated in NSCLC. Moreover, patients with higher expression of SOSTDC1 had a significant better prognosis than those with lower SOSTDC1 expression. Ectopic expression of SOSTDC1 in NSCLC cell lines A549 and NCI-H520 could inhibit proliferation as shown by MTT, colony formation, soft agar and EdU incorporation assays in vitro. Furthermore, A549 cells stably expressing ectopic SOSTDC1 grew more slowly and formed smaller tumors than vector-control cells in vivo. Mechanistic studies demonstrated that SOSTDC1 over-expression led to increased p21Cip and p27Kip levels, thereby decreasing Rb phosphorylation status and E2F transcription activity.

**Conclusions:**

SOSTDC1 is down-regulated in NSCLC, and its expression level is indicative of clinical outcome of patients with the disease. SOSTDC1 might represent a tumor suppressor through inhibiting the proliferation of NSCLC cells by regulating p21Cip and p27Kip, which in turn affects Rb-E2F signaling.

## Background

Lung cancer is one of the most commonly diagnosed cancer types worldwide and the leading cause of cancer-related death [1, 2]. Non-small cell lung cancer (NSCLC), which includes squamous cell carcinoma (SCC), adenocarcinoma (AD), large cell carcinoma (LCC), and other less frequently diagnosed histological types, accounts for about 80 % of lung cancer cases [3]. While various therapeutic approaches, including surgical resection, chemo- and radio-therapies, have been applied in the management of NSCLC, the overall 5-year survival rate of NSCLC patients still remains at only 15 % [4]. Better understanding of the genetic events and key molecules involved in NSCLC development and progression is needed for developing effective therapeutic strategies against the disease.

Sclerostin domain-containing protein 1 (SOSTDC1), an important regulator of cell signaling, has been found to contribute to several physiological and pathological processes [5, 6]. Accumulating evidence has revealed that SOSTDC1 might act as a tumor suppressor in many cancers. In wilms tumor, SOSTDC1 is lost as a result of a 7p21 homozygous deletion, which led to accelerate angiogenesis and activation of Wnt signaling [7]. The expression of SOSTDC1 is down-regulated in gastric cancer, and ectopic over-expression of SOSTDC1 in gastric cancer cells suppressed cell proliferation, cell cycle progression and anchorage-independent growth [8]. Clausen et al. found that the expression of SOSTDC1 reduced in breast cancer and such a reduction correlated  to poor prognosis of patients with the disease [9]. They also demonstrated that SOSTDC1 interfered with the signaling mediated by the Wnt3a, BMP-2, and BMP-7 pathways in breast cancer cells [10]. Moreover, down-regulation of SOSTDC1 was observed in renal cancer, and SOSTDC1 could suppress the proliferation of renal cancer cells via regulating BMP and Wnt3a signaling [11]. Nevertheless, the expression pattern and biological significance of SOSTDC1 in NSCLC remains largely unknown.

Deregulation of cell cycle progression represents a key characteristic in many types of cancer [12]. Under physiologically normal conditions, the cell cycle progression is elegantly regulated by cyclins and cyclin-dependent kinases (CDK) [13]. Of note, the activity of these molecules is restricted by the Cip/Kip family members [14–16], among which p21Cip and p27Kip are key functional cell cycle inhibitors containing an N-terminal CDK inhibitory domain and prevent CDKs from phosphorylating Rb, thus restricting the E2F transcription activity [14–17]. Given that E2F is an essential transcription factor for the production of cell cycle regulatory genes, p21Cip and p27Kip induces G1 arrest. Moreover, p21Cip could directly interfere with DNA replication by inhibiting PCNA [18]. Therefore, p21Cip and p27Kip are widely recognized as tumor suppressors, and their inactivation is frequently observed in various types of cancer, including NSCLC [19, 20]. While functional inactivation of p21Cip and p27Kip occur frequently in cancer cells, the mechanisms involved in the reduction or loss of p21Cip and p27Kip functions remain to be further understood.

In the context of understanding the possible role of SOSTDC1 in NSCLC development and progression, our current study led to findings that the protein is down-regulated in NSCLC and might play a tumor-suppressive role.

## Results

### The expression of SOSTDC1 is down-regulated in NSCLC

**Fig. 1:**
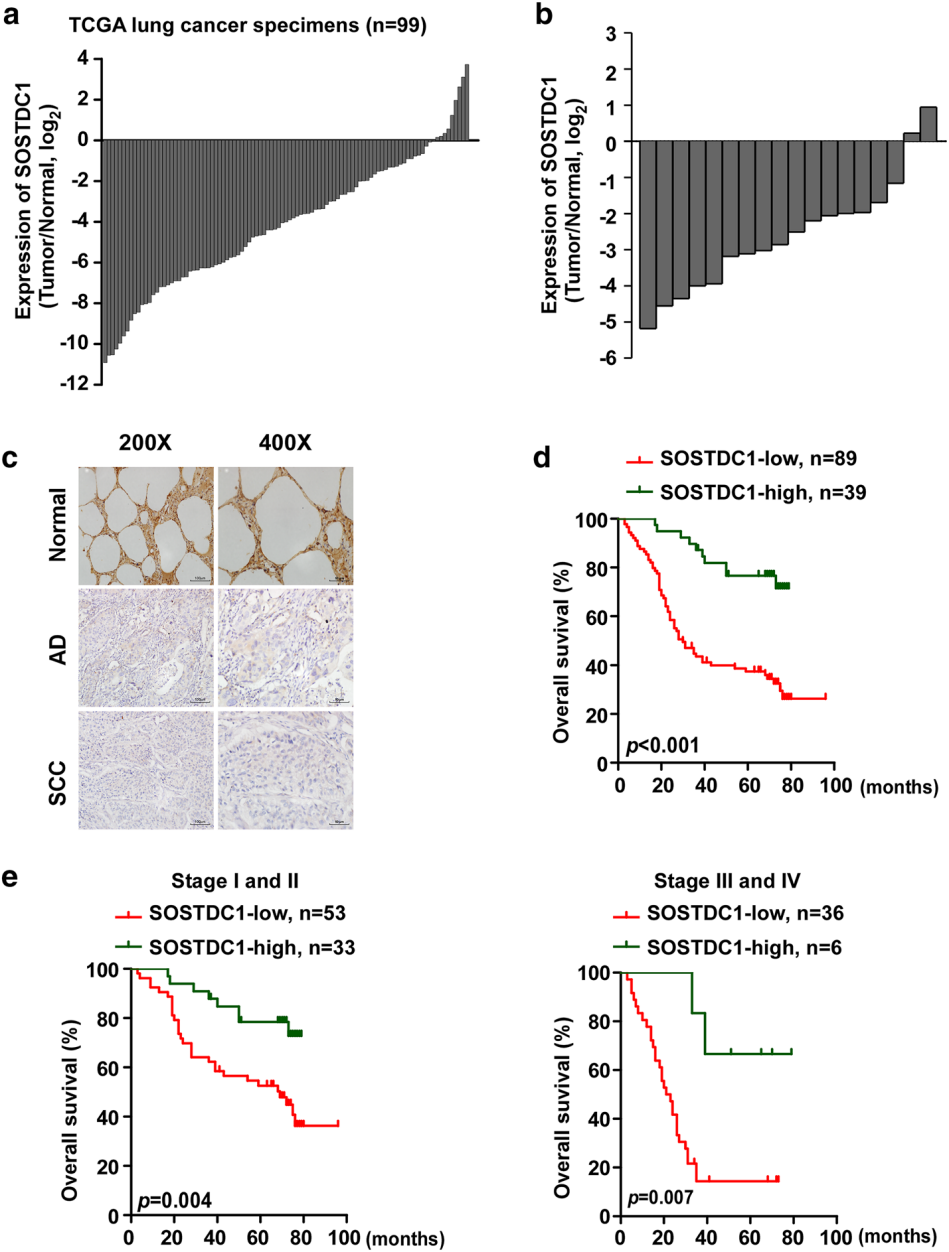
The expression of SOSTDC1 is down-regulated in NSCLC. **a** The expression of SOSTDC1 in 99 pairs of primary tumors versus paired non-tumorous lung tissues using RNAseqV2 data sets deposited in the TCGA datasets. **b** Expression of SOSTDC1 in 18 paired tumors and adjacent non-tumorous lung tissues assessed by qRT-PCR. **c** Representative images of IHC assays on SOSTDC1 expression in NSCLC. **d** Kaplan–Meier analysis showing the overall survival of NSCLC patients. **e** Kaplan–Meier analysis showing the overall survival of NSCLC patients categorized according to the UICC clinical stage. Patient survival is significantly different between SOSTDC1 high- and low-expressing patients within subgroups of clinical stage I + II and III + IV

To determine whether the expression of SOSTDC1 in NSCLC is altered, we first analyzed 99 pairs of primary tumor (T) versus normal tissues (N) using RNASeqV2 data sets for NSCLC deposited on the TCGA website (https://tcga-data.nci.nih.gov/tcga) [21] for SOSTDC1 expression. Our results showed that SOSTDC1 was down-regulated in 90 tumor tissues as compared to their adjacent non-cancerous lung tissues (Fig. 1a). To further assess the expression level of SOSTDC1 in NSCLC, we examined the level of SOSTDC1 in 18 pairs of tumors and their adjacent non-cancerous lung tissues. In agreement with the TCGA data, decrease in SOSTDC1 mRNA levels was observed in most tumor tissues as compared with that in their adjacent non-tumorous lung tissues (Fig. 1b). Next, we investigated the protein levels of SOSTDC1 in the 128 clinical specimens by performing immunohistochemistry (IHC) (Fig. 1c; Table 1). While no significant correlation between SOSTDC1 protein level and patient age (p = 0.732), gender (p = 0.150), pathologic type (p = 0.765), or N classification (p = 0.204) was found, the expression of SOSTDC1 significantly correlated to clinical staging (p = 0.010), and T classification (p < 0.001) (Table 2). Kaplan–Meier analysis using the log-rank test was performed to assay the effect of SOSTDC1 expression on the survival of NSCLC patients, and the data showed that patients with low SOSTDC1 expression had poorer overall survival than the high-SOSTDC1 expression group (p < 0.001) (Fig. 1d). Furthermore, the prognostic value of SOSTDC1 protein quantity in different subgroups of NSCLC patients stratified according to the clinical staging was also determined. As shown in Fig. 1e, low SOSTDC1 expression significantly correlated to shorter overall survival time in either the stage I–II subgroup (n = 86, p = 0.004, log-rank) or the stage III–IV subgroup (n = 42, p = 0.007, log-rank). Taken together, these data suggest that down-regulation of SOSTDC1 in NSCLC patients indicates a poorer prognosis.

**Table 1:** Clinicopathologic characteristics of patients enrolled in the study

	No of cases (%)
Age (y)	
≤56	66 (51.6)
>56	62 (48.4)
Gender	
Male	90 (70.3)
Female	38 (29.7)
Pathology	
Squamous cell carcinoma	44 (34.4)
Adenocarcinoma	76 (59.4)
Adenosquamous carcinoma	8 (6.2)
Clinical stage	
I	55 (43)
II	31 (24.2)
III	35 (27.3)
IV	7 (5.5)
T classification	
T1	27 (21.1)
T2	64 (50.0)
T3	33 (25.8)
T4	4 (3.1)
N classification	
N0	72 (56.2)
N1	31 (24.2)
N2	24 (18.8)
N3	1 (0.8)
Distant metastasis	
Yes	7 (5.5)
No	121 (94.5)

**Table 2:** Correlation between the clinical pathologic features and expression of SOSTDC1

Characteristics	SOSTDC1		*p* value
	Low	High	
Age (y)			
≤56	45	21	0.732
>56	44	18	
Gender			
Male	66	24	0.150
Female	23	15	
Pathologic type			
Squamous cell carcinoma	32	12	0.765
Adenocarcinoma	51	25	
Adenosquamous carcinoma	6	2	
Clinical staging			
I	30	25	0.010
II	23	8	
III	30	5	
IV	6	1	
T classification			
T1	10	17	<0.001
T2	46	18	
T3	29	4	
T4	4	0	
N classification			
N0	45	27	0.204
N1	23	8	
N2	20	4	
N3	1	0	

### Ectopic over-expression of SOSTDC1 inhibits the proliferation of NSCLC cells

**Fig. 2:**
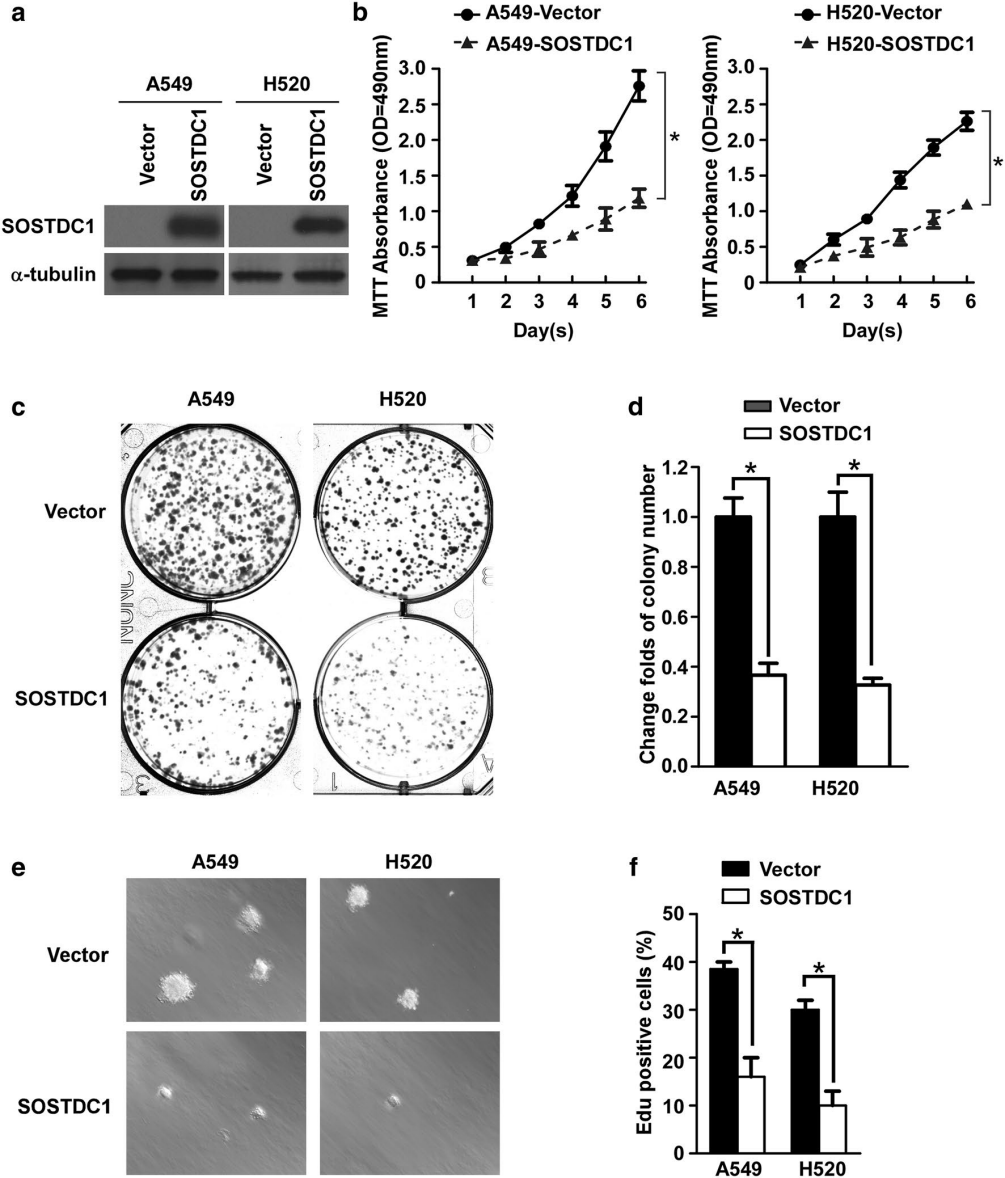
Ectopic over-expression of SOSTDC1 inhibits the proliferation of NSCLC cells. **a** Protein expression of SOSTDC1 in A549 and H520 cells was analyzed by Western blotting assay. α-tubulin was used as a loading control. **b** MTT assay was conducted to investigate the effect of SOSTDC1 on the proliferation of indicated cells at the indicated time points. **c** and **d** Representative micrographs (**c**) and relative quantification (**d**) of colony formation assays of indicated cells. **e** Representative images of anchorage-independent colonies formed by SOSTDC1-over-expressed cells. **f** Relative quantification of EdU incorporation assays. For **b**, **d**, and **f**, results are expressed as mean ± SD (n = 3), *p < 0.05

Next, we asked whether SOSTDC1 might play a role in the development and progression of the malignant phenotype of NSCLC cells. To this end, two NSCLC cell lines, including the lung adenocarcinoma cell line A549 and the lung squamous carcinoma cell line NCI-H520 (H520), were used. SOSTDC1 was ectopically over-expressed in A549 and H520 cells to generate stable cell lines, as confirmed by western blotting assay shown in Fig. 2a. As analyzed by MTT assay and shown in Fig. 2b, SOSTDC1 over-expression significantly repressed the cell viability of A549 and H520 cells, as compared with their corresponding control cells. Moreover, colony formation assay showed that the ability of both cell lines to form cellular colonies was significantly suppressed upon SOSTDC1 over-expression in comparison with that of their corresponding vector-control cells (Fig. 2c, d). Furthermore, we assessed the effect of high SOSTDC1 expression on anchorage independent growth using soft agar assay, and the results showed that cells expressing SOSTDC1 formed remarkably fewer and smaller colonies than the vector-control cells (Fig. 2e). In addition, EdU assays also revealed that the percentage of EdU-positive cell population was lower in SOSTDC1-over-expressed cells (Fig. 2f). The above data together indicate that SOSTDC1 over-expression significantly suppresses the proliferative ability of NSCLC cells.

### Ectopic over-expression of SOSTDC1 induces p21Cip and p27Kip expression and decreases the transcriptional activity of E2F

**Fig. 3:**
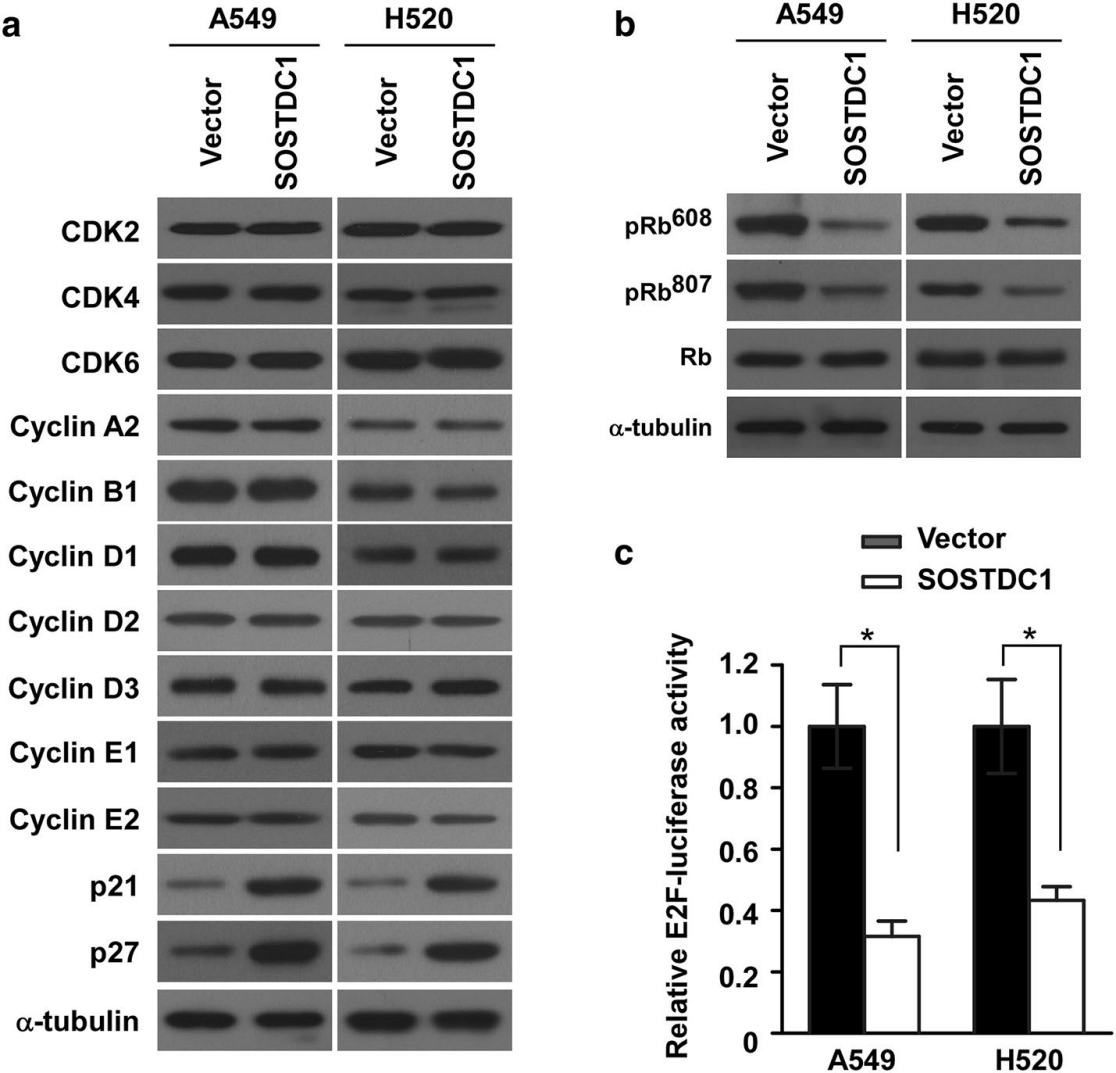
Ectopic over-expression of SOSTDC1 up-regulates the expression of p21Cip and p27Kip, and suppresses E2F transcriptional activity. **a** Western blotting analysis was performed to detect the cell cycle regulators CDK2, CDK4, CDK6, cyclin A2, cyclin B1, cyclin D1, cyclin D2, cyclin D3, cyclin E1, cyclin E2, p21Cip1 and p27Kip1 in indicated cells. α-tubulin was used as a loading control. **b** Ectopic expression of SOSTDC1 in the studied cells significantly inhibited the phosphorylation of pRb at Ser608 and Ser807 residues. α-tubulin served as the sample loading control. **c** Over-expression of SOSTDC1 attenuates E2F transcriptional activity using E2F-luc reporter assay. Results are expressed as mean ± SD (n = 3), *p < 0.05

To delineate the mechanism underlying the anti-proliferative effect of SOSTDC1 on NSCLC cells, the expression levels of cell cycle regulators were analyzed in SOSTDC1-over-expressing cells. As demonstrated in Fig. 3a, no alterations in the quantities of CDK2, CDK4, CDK6, cyclin A2, cyclin B1, cyclin D1, cyclin D2, cyclin D3, cyclin E1 and cyclin E2 were detected in NSCLC cell lines over-expressing ectopic SOSTDC1 when compared with the corresponding control cells. By contrast, the levels of both p21Cip1 and p27Kip1, two important CDKs inhibitors, significantly increased in SOSTDC1-transduced A549 and H520 cells. Moreover, ectopic expression of SOSTDC1 in NSCLC cells markedly inhibited the phosphorylation of Rb at Ser608 and Ser807 residues as well (Fig. 3b). As dephosphorylation of Rb is involved in regulating the transcriptional activity of E2F, we next tested whether SOSTDC1 could alter the transcriptional activity of E2F. As shown by our reporter assay illustrated in Fig. 3c, SOSTDC1 over-expression resulted in significant inhibition of the transactivating activity of E2F. Collectively, these data suggest that SOSTDC1 upregulates p21Cip and p27Kip expression and modulates Rb-E2F signaling.

### SOSTDC1 suppresses tumor growth in vivo

**Fig. 4:**
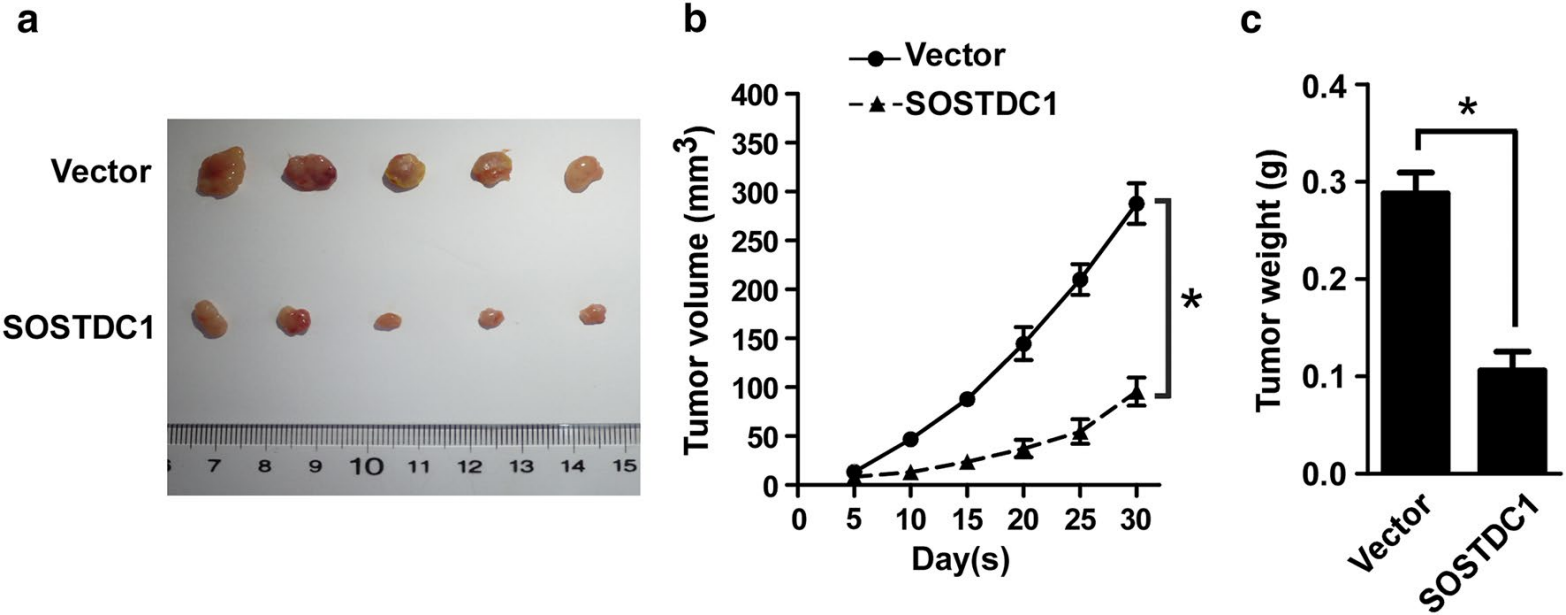
SOSTDC1 suppresses tumor growth in vivo. **a** Representative image of subcutaneous tumors isolated from nude mice. **b** Quantitative analysis of tumor volumes. **c** Quantitative analysis of tumor weights. The indicated tumor volumes and weights represent the mean ± SD of five animals in each group, *p < 0.05

The in vitro data that SOSTDC1 negatively regulates NSCLC cells proliferation prompted us to investigate whether SOSTDC1 can suppress tumorigenesis in vivo either. To this end, 8 × 10^6^ indicated NSCLC cells were subcutaneously inoculated into BALB/C nude mice (n = 5, respectively), and subcutaneous tumors volumes and weights were quantitatively analyzed. As shown in Fig. 4, over-expression of SOSTDC1 in NSCLC cells significantly inhibited tumor growth in vivo, further supporting the role of SOSTDC1 in NSCLC as a tumor suppressor gene.

## Discussion

In our current study, we find that SOSTDC1 expression is down-regulated in NSCLC, and that such an alteration of SOSTDC1 expression is associated with a poor clinical outcome of NSCLC patients. We also demonstrate that SOSTDC1 might inhibit the proliferation of NSCLC cells, possibly through up-regulating p21Cip and p27Kip and subsequent suppression of Rb-E2F signaling.

It has been well demonstrated that SOSTDC1 is involved in the process of fetal development [22–25]. Of note, accumulating studies have revealed that SOSTDC1 acts as a tumor suppressor in several types of cancers. For example, Clausen et al. found that expression of SOSTDC1 was down-regulated in breast cancer but a high SOSTDC1 expression correlates to better prognosis [10]. Furthermore, Blish et al. showed that SOSTDC1was expressed in human kidney tissues and significantly decreased in renal clear cell cancer [26]. Moreover, down-regulation of SOSTDC1 was also observed in thyroid cancer [27]. In NSCLC, several lines of evidence provided by our current study, including analysis of the public TCGA dataset, assessment of our fresh as well as archived clinical specimens and functional tests, has shown that SOSTDC1 is a down-regulated tumor suppressor in NSCLC cells.

Of note, various mechanisms have been proposed to contribute to the down-regulation of SOSTDC1 in cancers. For example, previous reports have shown hyper-methylation in SOSTDC1 promoter CpGs and epigenetic silencing of SOSTDC1 transcription via increased methylation in prostate cancer [9, 28]. Moreover, E4BP4, a transcriptional repressor, could bind to the promoter of SOSTDC1 gene and induce CpG hyper-methylation, thereby leading to repressed SOSTDC1 expression in breast cancer [8]. Intriguingly, genomic deletion also contributes to decreased SOSTDC1 expression. Specifically, a 7p21 homozygous deletion has been identified in 9 of 97 Wilms tumor, resulting in loss of SOSTDC1 [7], and loss of heterozygosity at 7p has also been reported to cause SOSTDC1 reduction in clear cell renal cell carcinoma [26]. Nevertheless, the exact molecular mechanism underlying the observed SOSTDC1 down-regulation in NSCLC is yet to be elucidated. Further studies are required to understand whether the aforementioned genomic and epigenetic alterations may also be responsible for the down-regulation of this important tumor suppressor in NSCLC, which are underway in our laboratory.

The observed down-regulation of SOSTDC1 in NSCLC implicates that SOSTDC1 is likely to be biologically involved in the development and progression of the disease. Indeed, our current data demonstrate that SOSTDC1 may be involved in regulating the proliferative capability of NSCLC cells, and that such an anti-proliferative function is associated with an upregulation of cell cycle-inhibitory factors p21Cip and p27Kip. In the light that p21Cip and p27Kip are both well recognized regulators of the RB-E2F signaling and indeed our present study has indicated a possible role of SOSTDC1 in modulating this cell-cycle regulatory pathway, it will be of great interest to further elucidate whether the observed changes of p21Cip and p27Kip is essential to the inhibitory effects of SOSTDC1 on cell proliferation. On the other hand, more systemic studies would be needed to explore whether other molecules or pathways are also involved in mediating the tumor-suppressive function of SOSTDC1 in NSCLC.

## Conclusions

SOSTDC1 is down-regulated and suppresses tumor growth in NSCLC. Down-regulation of SOSTDC1 is indicative of poor prognosis of the disease. The tumor suppressive function of SOSTDC1 is associated with upregulation of p21Cip and p27Kip, which may interfere with Rb-E2F signaling and disrupt the transactivating activity of E2F.

## Methods

### Cell cultures

Lung cancer cell lines, including A549 and H520, were obtained from American Type Culture Collection (ATCC), and maintained in DMEM medium (Invitrogen, Carlsbad, CA) supplemented with 10 % fetal bovine serum (HyClone, Logan, UT) and 1 % penicillin/streptomycin (Invitrogen, Carlsbad, CA), as previously reported [29]. The authenticity of the cell lines were verified by short tandem repeat fingerprinting at Medicine Laboratory of Forensic Medicine Department of Sun Yat-Sen University (Guangzhou, China).

### RNA extraction and real-time PCR

Total RNA from tissues was extracted by the TRizol reagent (Life Technologies, Gaithersburg, MD), and reverse transcription (RT) reactions and real-time polymerase chain reaction (PCR) were performed as described previously [29]. Primers were synthesized according to the following sequences: SOSTDC1 forward, 5′-CACGTTGAATCAAGCCAGAA-3′ and reverse, 5′-GATGTATTTGGTGGAACGCA-3′; and GAPDH forward, 5′-GACTCATGACCACAGTCCATGC-3′ and reverse, 5′-AGAGGCAGGGATGATGTTCTG-3′.

### Plasmids and transfection

SOSTDC1 expression plasmid was generated by PCR sub-cloning human SOSTDC1 coding sequence into retroviral transfer plasmid pQCXIP (Clontech, Palo Alto, CA) to generate plasmid pQCXIP-SOSTDC1. Retroviral production and infection were performed as we previously described, and stable cell lines were selected by treatment with 0.5 μg/ml puromycin for 10 days, beginning at 48 h after infection [27, 30].

### Western blotting analysis

Western blotting analysis was performed according to a standard method previously described [31], using anti-SOSTDC1, anti-cyclin B1, anti-cyclin D3 (Abcam, Cambridge, MA), anti-cyclin A2, anti-cyclin D1, anti-cyclin E1, anti-CDK4, anti-CDK6 (Epitomics, Burlingame, California), anti-cyclin D2, anti-CDK2 (BD Pharmingen, San Diego, CA), anti-cyclin E2, anti-p21Cip1, anti-p27Kip1, anti-p-Rb Ser608, anti-p-Rb Ser807, anti-Rb (Cell Signaling, Beverly, MA). When re-probing, blotted membranes were stripped and re-blotted with an anti-α-tubulin mouse monoclonal antibody (Sigma–Aldrich, St. Louis, MO) as a loading control.

### 3-(4,5-dimethyl-2-thiazolyl)-2,5-diphenyl-2-H-tetrazolium bromide (MTT) assay

According to previous studies [27], cell viability was determined using an MTT assay. The cells were seeded at a density of 5 × 10^3^ cells per well in 96-well plates. Subsequently at 1, 2, 3, 4, 5 and 6 days, 20 μl MTT (Sigma–Aldrich, St. Louis, MO) was added to each well and incubated for 4 h. The culture medium was removed, and 200 μl dimethyl sulfoxide (DMSO) (Amresco, Solon, Ohio) was added to each well. The plates were then shaken for 30 min, and the optical density (OD) at 490 nm was measured using a microplate reader. Each experiment was performed in triplicates.

### Colony formation assay

For the colony formation assay, cells were plated into 6-well plates at the density of 500 per well. The cells were allowed to grow for 10 days and stained with crystal violet. The plates were photographed and the numbers of colonies formed by indicated cells were quantified using the Quantity One software package (Bio-Rad, Hercules, CA). Each experiment was repeated for three times.

### Soft agar colony formation assay

Two milliliters of 0.66 % agar medium was added to each well of six-well plates to form bottom agar. Three thousand A549 cells and H520 cells were mixed with 2 ml of 0.33 % agar medium and then layered onto the bottom agar and incubated at 37 °C in 5 % CO_2_, and 0.5 ml of culture medium was added every week to keep the soft agar from drying and to supply nutrition. After 2 weeks of culture, the numbers of colonies were counted using a Zeiss microscope (Carl Zeiss, Jena, Germany).

### 5-ethynyl-2′-deoxyuridine (Edu) incorporation assa*y*

To examine the degree of DNA synthesis, the Cell Light EdU DNA imaging kit (RiboBio Co., Guangzhou, China) was used. Briefly, cells were seeded in 24-well plates and exposed to EdU for 2 h, followed by fixation in 4 % paraformaldehyde and permeabilization in 0.5 % Triton X-100. Images were taken using a fluorescent microscope at 488 nm excitation. Each experiment was repeated independently for three times.

### Luciferase reporter assay

The pE2F-TA-Luc reporter plasmid was purchased from the Clonetech Inc. (Mountain View, CA). Luciferase assay was performed as we previously described [32]. NSCLC cells were seeded in triplicate wells in 48-well plates and allowed to settle for 24 h. Two hundred nanogram of luciferase reporter plasmid or the control-luciferase plasmid, plus 5 ng of pRL-TK renilla plasmid (Promega, Madison, WI), was transfected into cells accompanied with indicated plasmid using the Lipofectamine 3000 reagent (Invitrogen, Carlsbad, CA) by following the manufacturer’s protocol. Dual Luciferase Reporter Assays were performed to test luciferase and renilla signals after 48 h according to a protocol provided with the Dual Luciferase Reporter Assay Kit (Promega, Madison, WI) by the manufacturer.

### Tumor specimens from patients

This study was conducted on a total of 128 paraffin-embedded NSCLC specimens, which were histopathologically and clinically diagnosed at the Sun Yat-Sen University Cancer Center from 2001 to 2006. The 18 NSCLC specimens and matched adjacent non-cancerous lung tissues were frozen and stored in liquid nitrogen until further use, according to our previous reports [29, 33]. For the use of these clinical materials for research purposes, prior patients’ consents and approval from the Institutional Research Ethics Committee were obtained.

### Immunohistochemistry assays (IHC)

IHC analysis was performed to study altered protein expression in human paraffin-embedded NSCLC specimens. The procedure was carried out similarly to previously described methods [34]. Immunostaining evaluations were performed independently by experimenters blinded to sample identity. The staining intensity was scored as follows: 0 (negative), 1 (weakly positive), 2 (moderately positive), and 3 (strongly positive). The percent positivity was also scored according to four categories 0 (<5 %), 1 (5–25 %), 2 (>25–50 %), 3 (>50–75 %) and 4 (>75 %). Then the value of percent positivity score was multiplied by staining intensity score to generate final expression scores of SOSTDC1, which ranged from 0 to 12. The staining scores were defined as follows: low expression (score ≤ 2) and high expression (score ≥ 3).

### In vivo tumorigenesis assay

Animal protocols were approved by the Ethical Committee of Sun Yat-sen University. BALB/c nude mice were injected sc with 8 × 10^6^ indicated cells in 0.1 ml of PBS in the right flank. Tumor volumes were measured with calipers and calculated by the formula: 0.52 × length in millimeters × (width in millimeters)^2^. Thirty days later, mice were killed, and tumors were excised and weighed.

### Statistical analysis

All statistical analyses were carried out using the SPSS 19. 0 statistical software package. The Kaplan–Meier method was used to establish survival curves, and log-rank test was applied for comparative analysis of differences in patient survival. All values represent at least three independent experiments and are expressed as the mean ± SD. Comparisons between groups for statistical significance were performed with a two-tailed paired Student’s t test. In all cases, *p* < 0. 05 was considered statistically significant.

## References

[1] Ferlay J, Soerjomataram I, Dikshit R, Eser S, Mathers C, Rebelo M (2015). Cancer incidence and mortality worldwide: sources, methods and major patterns in GLOBOCAN 2012. Int J Cancer.

[2] Siegel R, Ma J, Zou Z, Jemal A (2014). Cancer statistics, 2014. CA Cancer J Clin.

[3] Schiller JH, Harrington D, Belani CP, Langer C, Sandler A, Krook J (2002). Comparison of four chemotherapy regimens for advanced non-small-cell lung cancer. N Engl J Med.

[4] Spira A, Ettinger DS (2004). Multidisciplinary management of lung cancer. N Engl J Med.

[5] Yanagita M, Oka M, Watabe T, Iguchi H, Niida A, Takahashi S (2004). USAG-1: a bone morphogenetic protein antagonist abundantly expressed in the kidney. Biochem Biophys Res Commun.

[6] Laurikkala J, Kassai Y, Pakkasjarvi L, Thesleff I, Itoh N (2003). Identification of a secreted BMP antagonist, ectodin, integrating BMP, FGF, and SHH signals from the tooth enamel knot. Dev Biol.

[7] Ohshima J, Haruta M, Arai Y, Kasai F, Fujiwara Y, Ariga T (2009). Two candidate tumor suppressor genes, MEOX2 and SOSTDC1, identified in a 7p21 homozygous deletion region in a Wilms tumor. Genes Chromosomes Cancer.

[8] Rawat A, Gopisetty G, Thangarajan R (2014). E4BP4 is a repressor of epigenetically regulated SOSTDC1 expression in breast cancer cells. Cell Oncol (Dordr).

[9] Gopal G, Raja UM, Shirley S, Rajalekshmi KR, Rajkumar T (2013). SOSTDC1 down-regulation of expression involves CpG methylation and is a potential prognostic marker in gastric cancer. Cancer Genet.

[10] Clausen KA, Blish KR, Birse CE, Triplette MA, Kute TE, Russell GB (2011). SOSTDC1 differentially modulates Smad and beta-catenin activation and is down-regulated in breast cancer. Breast Cancer Res Treat.

[11] Blish KR, Wang W, Willingham MC, Du W, Birse CE, Krishnan SR (2008). A human bone morphogenetic protein antagonist is down-regulated in renal cancer. Mol Biol Cell.

[12] Malumbres M, Barbacid M (2009). Cell cycle, CDKs and cancer: a changing paradigm. Nat Rev Cancer.

[13] Satyanarayana A, Kaldis P (2009). Mammalian cell-cycle regulation: several Cdks, numerous cyclins and diverse compensatory mechanisms. Oncogene.

[14] Guan KL, Jenkins CW, Li Y, Nichols MA, Wu X, O’Keefe CL (1994). Growth suppression by p18, a p16INK4/MTS1- and p14INK4B/MTS2-related CDK6 inhibitor, correlates with wild-type pRb function. Genes Dev.

[15] Sotillo R, Renner O, Dubus P, Ruiz-Cabello J, Martin-Caballero J, Barbacid M (2005). Cooperation between Cdk4 and p27kip1 in tumor development: a preclinical model to evaluate cell cycle inhibitors with therapeutic activity. Cancer Res.

[16] Kato JY, Matsuoka M, Polyak K, Massague J, Sherr CJ (1994). Cyclic AMP-induced G1 phase arrest mediated by an inhibitor (p27Kip1) of cyclin-dependent kinase 4 activation. Cell.

[17] Kiyokawa H, Kineman RD, Manova-Todorova KO, Soares VC, Hoffman ES, Ono M (1996). Enhanced growth of mice lacking the cyclin-dependent kinase inhibitor function of p27(Kip1). Cell.

[18] Luo Y, Hurwitz J, Massague J (1995). Cell-cycle inhibition by independent CDK and PCNA binding domains in p21Cip1. Nature.

[19] Mitchell KO, El-Deiry WS (1999). Overexpression of c-Myc inhibits p21WAF1/CIP1 expression and induces S-phase entry in 12-O-tetradecanoylphorbol-13-acetate (TPA)-sensitive human cancer cells. Cell Growth Differ.

[20] Masciullo V, Sgambato A, Pacilio C, Pucci B, Ferrandina G, Palazzo J (1999). Frequent loss of expression of the cyclin-dependent kinase inhibitor p27 in epithelial ovarian cancer. Cancer Res.

[21] Cancer Genome Atlas Research N (2008). Comprehensive genomic characterization defines human glioblastoma genes and core pathways. Nature.

[22] Cho SW, Kwak S, Woolley TE, Lee MJ, Kim EJ, Baker RE (2011). Interactions between Shh, Sostdc1 and Wnt signaling and a new feedback loop for spatial patterning of the teeth. Development.

[23] Collette NM, Yee CS, Murugesh D, Sebastian A, Taher L, Gale NW (2013). Sost and its paralog Sostdc1 coordinate digit number in a Gli3-dependent manner. Dev Biol.

[24] Ahn Y, Sims C, Logue JM, Weatherbee SD, Krumlauf R (2013). Lrp4 and Wise interplay controls the formation and patterning of mammary and other skin appendage placodes by modulating Wnt signaling. Development.

[25] Clavel C, Grisanti L, Zemla R, Rezza A, Barros R, Sennett R (2012). Sox2 in the dermal papilla niche controls hair growth by fine-tuning BMP signaling in differentiating hair shaft progenitors. Dev Cell.

[26] Blish KR, Clausen KA, Hawkins GA, Garvin AJ, Willingham MC, Turner JC (2010). Loss of heterozygosity and SOSTDC1 in adult and pediatric renal tumors. J Exp Clin Cancer Res.

[27] Liang W, Guan H, He X, Ke W, Xu L, Liu L (2015). Down-regulation of SOSTDC1 promotes thyroid cancer cell proliferation via regulating cyclin A2 and cyclin E2. Oncotarget.

[28] Tesfay L, Clausen KA, Kim JW, Hegde P, Wang X, Miller LD (2015). Hepcidin regulation in prostate and its disruption in prostate cancer. Cancer Res.

[29] Yang Y, Liu L, Zhang Y, Guan H, Wu J, Zhu X (2014). MiR-503 targets PI3K p85 and IKK-beta and suppresses progression of non-small cell lung cancer. Int J Cancer.

[30] Yang Y, Liu L, Cai J, Wu J, Guan H, Zhu X (2014). DEPDC1B enhances migration and invasion of non-small cell lung cancer cells via activating Wnt/beta-catenin signaling. Biochem Biophys Res Commun.

[31] Cai J, Guan H, Fang L, Yang Y, Zhu X, Yuan J (2013). MicroRNA-374a activates Wnt/beta-catenin signaling to promote breast cancer metastasis. J Clin Invest.

[32] Zhu X, He Z, Hu Y, Wen W, Lin C, Yu J (2014). MicroRNA-30e* suppresses dengue virus replication by promoting NF-kappaB-dependent IFN production. PLoS Negl Trop Dis.

[33] Cai J, Wu J, Zhang H, Fang L, Huang Y, Yang Y (2013). miR-186 downregulation correlates with poor survival in lung adenocarcinoma, where it interferes with cell-cycle regulation. Cancer Res.

[34] Jiang L, Lin C, Song L, Wu J, Chen B, Ying Z (2012). MicroRNA-30e* promotes human glioma cell invasiveness in an orthotopic xenotransplantation model by disrupting the NF-kappaB/IkappaBalpha negative feedback loop. J Clin Invest.

